# Integrative Analysis of RNA Expression and Regulatory Networks in Mice Liver Infected by *Echinococcus multilocularis*

**DOI:** 10.3389/fcell.2022.798551

**Published:** 2022-03-24

**Authors:** Tingli Liu, Hong Li, Yanping Li, Liqun Wang, Guoliang Chen, Guiting Pu, Xiaola Guo, William C. Cho, Majid Fasihi Harandi, Yadong Zheng, Xuenong Luo

**Affiliations:** ^1^ State Key Laboratory of Veterinary Etiological Biology, Key Laboratory of Veterinary Parasitology of Gansu Province, Lanzhou Veterinary Research Institute, CAAS, Lanzhou, China; ^2^ Department of Clinical Oncology, Queen Elizabeth Hospital, Hong Kong SAR, China; ^3^ Research Center for Hydatid Disease in Iran, Kerman University of Medical Sciences, Kerman, Iran; ^4^ Key Laboratory of Applied Technology on Green-Eco-Healthy Animal Husbandry of Zhejiang Province, Zhejiang International Science and Technology Cooperation Base for Veterinary Medicine and Health Management, Zhejiang Provincial Engineering Laboratory for Animal Health Inspection and Internet Technology, Zhejiang International Science and Technology Cooperation Base for Veterinary Medicine and Health Management, China-Australia Joint Laboratory for Animal Health Big Data Analytics, College of Animal Science and Technology and College of Veterinary Medicine of Zhejiang A&F University, Hangzhou, China

**Keywords:** *Echinococcus multilocularis*, hepatic cells, hepatic stellate cells, kupffer cells, regulatory network

## Abstract

The larvae of *Echinococcus multilocularis* causes alveolar echinococcosis, which poses a great threat to the public health. However, the molecular mechanisms underlying the host and parasite interactions are still unclear. Exploring the transcriptomic maps of mRNA, miRNA and lncRNA expressed in the liver in response to *E. multilocularis* infection will help us to understand its pathogenesis. Using liver perfusion, different cell populations including the hepatic cells, hepatic stellate cells and Kupffer cells were isolated from mice interperitoneally inoculated with protoscoleces. Their transcriptional profiles including lncRNAs, miRNAs and mRNAs were done by RNA-seq. Among these cell populations, the most differentially-expressed (DE) mRNA, lncRNAs and miRNAs were annotated and may involve in the pathological processes, mainly including metabolic disorders, immune responses and liver fibrosis. Following the integrative analysis of 38 differentially-expressed DEmiRNAs and 8 DElncRNAs, the lncRNA-mRNA-miRNA networks were constructed, including F63-miR-223-3p-Fbxw7/ZFP36/map1b, F63-miR-27-5p-Tdrd6/Dip2c/Wdfy4 and IFNgAS1-IFN-γ. These results unveil the presence of several potential lncRNA-mRNA-miRNA axes during *E. multilocularis* infection, and further exploring of these axes may contribute to better understanding of the pathogenic mechanisms.

## Introduction

Alveolar echinococcosis (AE), a zoonosis with an increasing concern, is caused by a canine parasite *Echinococcus multilocularis* (Eckert and Deplazes, 2004). At which human beings are infected by intake of food or water contaminated with the eggs shed by definitive hosts, such as dogs and foxes (Conraths et al., 2017; Liu et al., 2018; Wang et al., 2018; Kotwa et al., 2019). Once infected, the eggs penetrate the small intestine wall, finally reach the liver and lung and then grow in an infiltrative pattern (Czermak et al., 2008; Mueller et al., 2020). The clinical manifestations of human AE are diverse, including jaundice, weight loss, fever, anaemia, and abdominal pain (Kolářová et al., 2015; Massolo et al., 2019).

The main treatment strategies for AE are surgery and anti-echinococcal drugs, such as albendazole (ABZ) and mebendazole (MBZ) (Craig et al., 2007; Gottstein et al., 2015; Wang S. et al., 2021; Huang et al., 2021). However, both strategies have limitations. For surgical treatment, it is prone to recurrent due to diffuse and undetected parasite infiltration into host tissues (Huang et al., 2021). In terms of drug treatment, the poor intestinal absorption and some side effects are concerned (Gottstein et al., 2015; Craig et al., 2017). Some other treatment strategies such as traditional Chinese medicines and new formulation drug have limited treatment efficacy (Maggiore et al., 2015; Almalki et al., 2017; Pensel et al., 2017; Torabi et al., 2018). Therefore, it is urgent to study the genome-scale transcriptional background of the liver in response to the infection for discovering the potential molecular target for the effective therapeutics.

lncRNAs, with the length of more than 200 bp, have been confirmed as a momentous regulatory factor in many cellular processes, such as genomic imprinting, post transcriptional regulation, and cell differentiation (Kallen et al., 2013; Myant et al., 2013; Yang Q. et al., 2018). lncRNAs show a competing endogenous effect by acting as miRNA sponges, which is helpful to decipher the miRNA-lncRNA regulatory networks (Li et al., 2020; Zheng et al., 2020). Another non-coding RNAs, miRNAs, are members of endogenous small RNA with the length of about 18–24 nucleotides, involved in the regulation of mRNA expression. By binding to the 3′-untranslated region, miRNAs block the translation or induce the degradation of the target mRNA, thus modulating cell differentiation, growth, proliferation and apoptosis (Li et al., 2021; Mohammadi et al., 2021; Xiang et al., 2021). miRNAs have been reported as diagnostic markers and therapeutic targets for the control of diseases (Broermann et al., 2020; Tombolan et al., 2020). For instance, miR-155 was exploited as the potential target for treatment of the disease by *Toxoplasma gondii* (Xu et al., 2021). Another study found that the novel-miR-1 derived from *Cysticercus pisiformis* was released into host serum, which could be exploited for diagnosis of *C. pisiformis* infection (Chen et al., 2021).

Clinically, the intrahepatic lesions are the most common clinical trait of AE (Woolsey and Miller, 2021). The liver is comprised of a number of specialized cells, e,g, hepatocytes (HCs) as the hepatic parenchyma are the most abundant cell population, performing the fundamental functions in endocrine homeostasis and metabolism (Wang et al., 2015). Besides, there is a small proportion of nonparenchymal cells (NPCs), including hepatic stellate cells (HSCs), Kupffer cells (KCs), and hepatic sinusoidal endothelial cells (LSECs). Among these cells, HSCs reside in the perisinusoidal space (space of Disse) filled with thin permeable connective tissues, which have been recognized as the major source of type I and III collagens and fibronectin. Under normal condition, quiescent HSCs (qHSCs) are characterized by enrichment of vitamin A in cytoplasm, responsible for tissue homeostasis by involving in proliferation and differentiation signaling (Balmer and Blomhoff. 2002). Upon stimuli, they transfer into activated HSCs (aHSCs) that secret a-SMA and extracellular matrix (ECM) components (type I and III collagens and fibronectin). As a result, the persisting increase of ECM components will trigger liver fibrosis, possibly leading to liver cancer. However, once the stimuli disappear, the aHSCs gradually return back to the quiescent ones and the degree of fibrosis decreases (Kisseleva et al., 2012; Khomich et al., 2019). Another important component of NPCs, KCs, is the major immune cells that reside in the liver, responsible for clearing foreign materials under the normal conditions. Moreover, KCs protect liver from injury by releasing cytokines, reactive oxygen and others, playing a key role in the acute and chronic responses in the liver injury (Roberts et al., 2007).

Various liver cell populations perform distinct biological functions, and thus it is important to elucidate their role during *E. multilocularis* infection. In this study, we defined the transcriptomes of HCs, HSCs and KCs at two time points (2 and 3 months) post infection (p.i.) of *E. multilocularis*. Besides, the potential interaction networks among differentially expressed mRNAs, miRNAs and lncRNAs were identified. The current results provide the transcriptional expression of three liver cell populations in response to *E. multilocularis* infection and a clue for further investigation of a role of the networks in the pathogenesis.

## Materials and Methods

### Parasites

The protoscolices used in this study were obtained from *Mongolian gerbil* infected with *E. multilocularis* as previously described (Spiliotis and Brehm, 2009). In brief, cysts were dissected from infected *Mongolian gerbil* under sterile conditions. After cutting the cysts into pieces, the protoscolices were collected by gravity and washed several times in cold PBS. The purity and activity of protoscolices were checked using optical microscopy and trypan blue exclusion, respectively.

### Animal Infection

100 six-week old BALB/c mice were purchased from Laboratory Animal Center of Lanzhou Veterinary Research Institute and were randomly divided two groups: experimental (E) group (60 mice) and control (C) group (40 mice). In E group, 600 protoscolices were injected into the abdominal cavity. In C group, the same volume of phosphate buffer saline (PBS) was injected. In E group, whose cysts in the liver were considered as infected, otherwise not. In order to obtain enough cells at two timepoints 2 m p.i. and 3 m p.i., by the cause of a few HSCs and KCs in the liver, we mixed cells from six mice for RNA-seq and qRT-PCR, and three batches of samples were concluded in two groups. All mice were reared under standard feeding conditions, free access to food and water.

### Isolation of HCs, HSCs and KCs

We collected the HCs, HSCs and KCs samples from mice 2 m p.i. and 3 m p.i. in E and C groups, accordingly named as HC-2M-E, HC-2M-C, HC-3M-E, HC-3M-C, HSC-2M-E, HSC-2M-C, HSC-3M-E, HSC-3M-C, KC-2M-E, KC-2M-C, KC-3M-E and KC-3M-C. In each group, the cells of six mice were mixed together to obtain enough HSCs and KCs. The perfusion procedure for mouse liver was strictly followed as previously reported (Mederacke et al., 2015). In detail, 10 ml of 0.019% EGTA and 30 ml of 0.04% collagenase Ⅳ solution were sequentially perfused into every liver, followed by further liver digestion using 80 ml of 0.08% collagenase Ⅳ solution with 1% DNase. The digested liver tissues were filtered into a 50 ml tube through a 70 μm cell strainer. The mixture was centrifuged at 50 ×*g* for 4 min at 4°C, and the cell pellet was washed three times in the DMEM with 5% FBS to obtain HCs. The supernatant was sequentially centrifuged at 600 ×*g* and 500 ×*g* for 10 min at 4°C to obtain NPCs. The NPC-containing solution was mixed with 5 ml Gey’s Balanced Salt Solution (GBSS), then the mixture was gently overlaid onto the Optiprep solutions at different concentrations, which contained 8 ml of 11.5% Optiprep in the upper layer and 4 ml of 20% Optiprep in the bottom, followed by centrifugation at 1,400 ×*g* for 17 min at 4°C without break. The cells in the upper layer (HSCs) and the lower layer (KCs and ECs) were transferred into two centrifuge tubes, respectively, and washed three times in GBSS. Then, KCs and ECs were cultured in RPMI 1640 medium with 10% fetal bovine serum (FBS). After 4 h, KCs were adhered completely and collected after 0.25% trypsin digestion, while ECs were not adhered and abandoned after PBS washed (Zheng et al., 2008). The HCs, HSCs and KCs were immediately stored at −80°C for later use.

### RNA-Seq

Following the manufacturer’s instructions, total RNA was extracted using TRIzol reagent. After evaluation of RNA concentration and integrity, these samples were used to construct the libraries for sequencing (BGI, Wuhan, China). For construction of the lncRNA + mRNA library, rRNA-depleted RNA was fragmented by adding first strand master mix (Invitrogen, United States), and the first-strand and second-strand cDNA was generated separately by using random primers reverse transcription. Then, the cDNA was subjected to end-repair and was 3′ adenylated, the adapters were ligated to the end of 3′ adenylated cDNA. After PCR amplification, the cDNA fragments (lncRNA + mRNA library) were enriched and purified with Ampure XP beads.

For small RNA library, the 18–30 nt bands were excised and recovered from total RNA by using 15% urea denaturing polyacrylamide gel electrophoresis (PAGE) gel. Subsequently, the 18–30 nt small RNAs were ligated to adenylated 3′ and 5′ adapters separately and transcripted into cDNA by SuperScript Reverse Transcriptase (Invitrogen, United States). After PCR amplification, the cDNA fragments was enriched and 110–130 nt fragments (small RNA library) were selected by agarose gel electrophoresis. Last, the BGISEQ-500 platform (BGI, Wuhan, China) was used to sequence. In order to get clean reads, the reads with low quality and adaptor contaminants were removed from the raw data using SOAPnuke software (v1.5.2; -l 15 -q 0.2 -n 0.05) and the Q20, Q30 and GC content were calculated to assess the quality of the clean reads.

### Data Analysis

Using HISAT2 software (v2.0.4; http://www.ccb,jhu.edu/software/hisat/index.shtml), the clean reads were aligned against the *Mus musculus* genome (GCF_000001635.26_GRCm38.p6). Bowtie2 (v2.2.5; http://bowtiebio.sourceforge.net/%20Bowtie2%20/index.shtml) was used to align the clean reads to known and novel, coding and noncoding transcripts. Subsequently, the expression levels of mRNA and lncRNAs were calculated by using RESM (v1.2.12; http://github.com/deweylab/RSEM) with the FPKM standardized method. After analyzing the results of the relative expression of genes in this study, we found the genes was mainly enriched near 2 in fold change. Besides, we used multiple hypothesis test correction for the *p*-value of the difference test, and False Discover Rate (FDR) ≤ 0.001 was considered as statistically different. Based on these, the differentially expressed lncRNAs (DElncRNAs) and differentially expressed mRNA (DEmRNAs) with a fold change ≥2 and FDR ≤0.001 were screened out.

### Functional Enrichment Analysis

We predicted the potential targets of DElncRNAs by analyzing the position between genes in the genome as previously reported (Ren et al., 2018). If the relative position between genes was less than 10 kb, we defined it as cis-regulation. Otherwise, we defined it as trans-regulation. Besides, for constructing the lncRNA-mRNA-miRNA networks, target prediction of miRNAs to the DEmRNAs and DElncRNAs was performed using RNAhybrid, miRanda and TargetScan databases.

Gene Ontology (GO) was used to describe the genetic attributes with terms under the biological process, cellular component, and molecular function categories. By comparing the DElncRNAs, DEmRNAs and the targets of DEmiRNAs with the genes of mouse, the significantly enriched GO terms were determined. Similarly, the significantly enriched pathway terms were obtained using the KEGG pathway database. The GO and pathway terms with Q value ≤0.05 was defined as a term which was significantly enriched.

### qRT-PCR Assay

For evaluation of the purity of liver cell populations and the expression of DEmRNAs, DElncRNAs and DEmiRNAs, qRT-PCR was performed. The genes for the cell markers (Alb, F4/80, α-SMA, Col1α1, Col3α1 and GFAP), DElncRNAs and their nearby genes located within the 10 kb upstream and downstream in the genome, and the metabolism- and inflammation-related DEmRNAs were selected for validation. 1 μg of total RNA was reversely transcribed to cDNA using HiScript Ⅲ First-strand cDNA Synthesis Kit (Vazyme, Munich, Germany) as recommended by the manufacturer. qRT-PCR was performed using All-in-One^TM^ qPCR Mix (GeneCopoeia) with an ABI 7500 Thermal Cycler (ThermoFisher Scientific, United States) according to the standard method. Specific primers for the selected DElncRNAs and DEmRNAs were obtained from TSINGKE (Xi’an, China) (Supplementary Table S1), and specific primers for miRNAs were purchased from GeneCopoeia (United States). The relative expression level was normalized to GAPDH for DEmRNAs and DElncRNAs or to U6 for DEmiRNAs. The 2^−ΔΔCt^ algorithm was used to calculate the relative expression levels represented as relative fold-change (FC). All experiments were performed in triplicate.

### Statistical Analysis

The statistical analysis was conducted using Prism 6 (GraphPad) software. The comparison between E and C groups was analyzed using Student’s *t*-test. The results were presented as mean ± standard deviation (SD). Significant differences were indicated as *p < 0.05, **p < 0.01 and ***p < 0.001.

## Results

### Primary HC, HSC and KC Isolation and Characterization

The primary HCs, HSCs and KCs were isolated from liver of anesthetized mice by using 0.04% collagenase Ⅳ solution, with the largest number of HCs, followed by KCs and then HSCs. By comparing the relative expression levels of cell markers (HCs: Alb, HSCs: α-SMA, GFAP, Col1α1 and Col1α3; KCs: F4/80), the results showed that Alb was predominantly expressed in HCs, with the percentage of 69.44, F4/80 predominantly expressed in KC with 73.19, and α-SMA, GFAP, Col1α1 and Col1α3 predominantly expressed in HSC (Supplementary Figure S1) with 83.09, 87.70, 93.36 and 89.55, respectively, suggesting each type of cell was enrichment.

### Sequencing Data

As shown in Table 1, after removal of the low quality reads, 112M–115M total reads were produced in mRNA + lncRNA libraries and 24–25M in miRNA libraries. In mRNA + lncRNA libraries, the mapped percent was ranged from 95 to 96 with the average mapped rate of 96% and a total of 43,183 genes were identified. In miRNA libraries, 88–96% clean reads were mapped with the average mapped rate of 93% and a total of 1,201 small RNAs were predicted. Additionally, a total of 33,065 genes were detected in HCs, 37,637 in HSCs and 39,174 in KCs.

**TABLE 1:** The statistics of sequencing data.

Cell	Genes	Group	2 m p.i.						3 m p.i.					
			mRNA + lncRNA			miRNA			mRNA + lncRNA			miRNA		
			Total reads (M)	Clean reads (M)	Mapped percent (%)	Total reads (M)	Clean reads (M)	Mapped percent (%)	Total reads (M)	Clean reads (M)	Mapped percent (%)	Total reads (M)	Clean reads (M)	Mapped percent (%)
HC	33,065	Control	112.4	111.2	96.2	25.2	23.6	91.6	111.9	113.9	96.3	23.7	22.1	89.9
		Infected	112.4	111.7	95.9	25.2	24.1	93.4	111.9	113.9	96.3	25.2	23.6	88.2
HSC	37,637	Control	114.9	113.7	96.3	25.2	24.5	94.8	114.9	113.8	95.6	25.2	24.6	92.1
		Infected	112.4	111.8	96.0	25.2	24.5	92.6	114.9	113.8	96.0	25.2	24.6	94.4
KC	39,174	Control	112.4	111.7	94.9	25.2	24.6	95.7	114.9	113.8	95.0	25.2	24.6	94.5
		Infected	112.4	111.8	95.0	25.2	24.6	95.9	112.4	111.4	95.3	25.2	24.5	94.4

### LncRNA, mRNA and miRNA Profiles in HCs, HSCs and KCs

**FIGURE 1:**
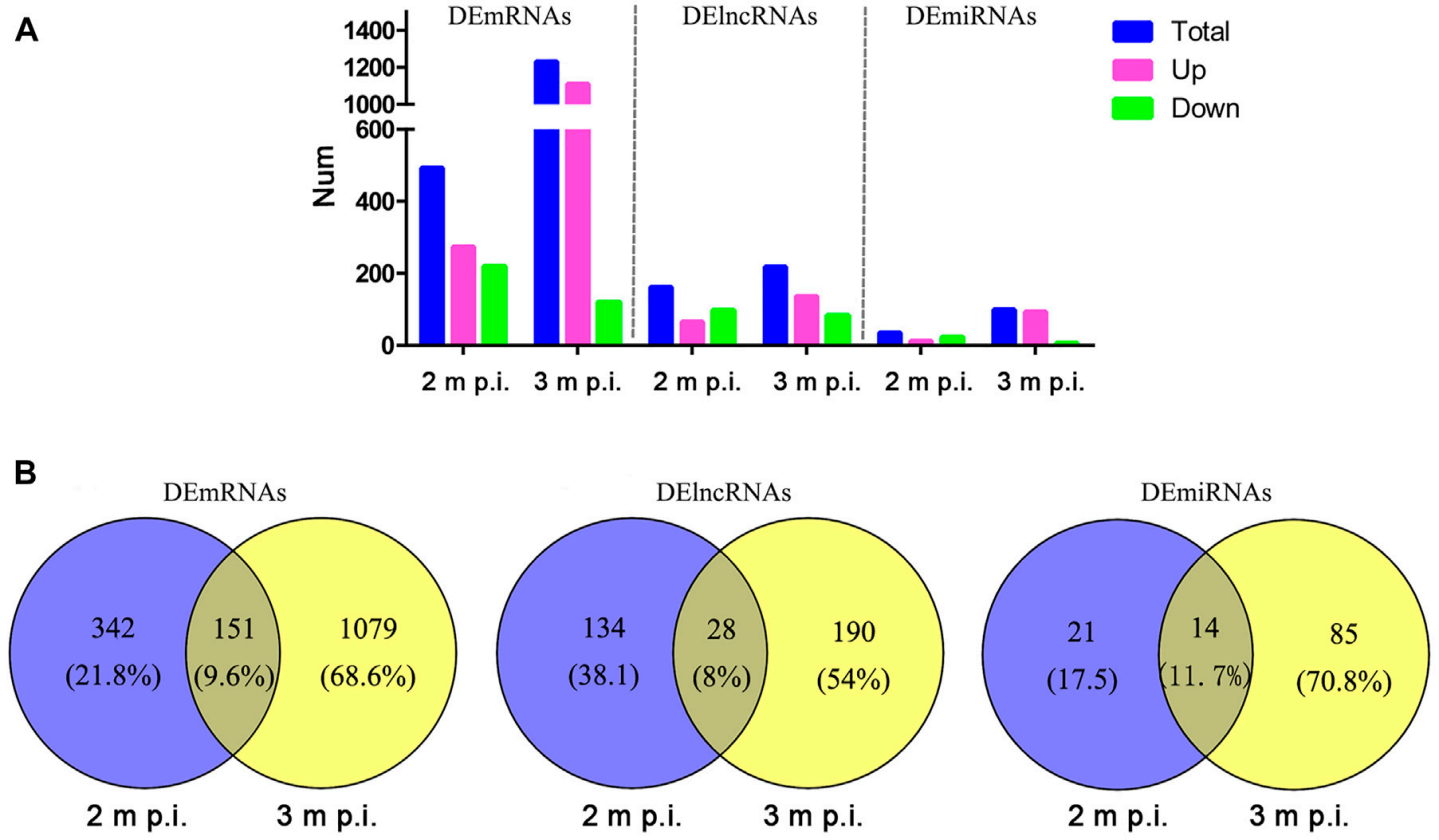
Comparisons of DElncRNAs, DEmRNAs and DEmiRNAs differentially expressed in HCs at 2 m p.i. and 3 m p.i **(A)** The number of DElncRNAs, DEmRNAs and DEmiRNAs at two infection stages. Sky blue represents the total number of DEgemes, red and violet represents upregulated and downregulated genes, respectively. **(B)** Veen diagrams showing the common and unique DElncRNAs, DEmRNAs and DEmiRNAs at two infection stages.

As shown in Figure 1, in 2 m p.i. HC samples, 274 upregulated and 219 downregulated mRNA were identified. Simultaneously, 1,111 upregulated and 119 downregulated mRNA were identified in 3 m p.i. samples. Additionally, 65 upregulated and 97 downregulated lncRNAs, and 12 upregulated and 23 downregulated miRNAs were found in 2 m p.i. samples, while 136 upregulated and 82 downregulated lncRNAs, and 93 upregulated and 6 downregulated miRNAs in 3 m p.i. samples (Figure 1A, Supplementary Table S2). Furthermore, DEmRNAs, DElncRNAs and DEmiRNAs of two time point were identified, with the commonly shared 151 DEmRNAs (9.6%), 28 DElncRNAs (8%) and 14 DEmiRNAs (11.7%) (Figure 1B).

In 2 m p.i. HSC samples, total 293 upregulated and 335 downregulated mRNAs, 98 upregulated and 127 downregulated lncRNAs, and 11 upregulated and 20 downregulated miRNAs were found, while total 241 upregulated and 142 downregulated mRNAs, 66 upregulated and 63 downregulated lncRNAs, and 56 upregulated and 21 downregulated miRNAs were found in 3 m p.i. HSC samples (Supplementary Figure S2A, Supplementary Table S2). The 80 (8.6%), 22 (6.6%) and 18 (20%) common differentially-expressed genes (DEGs) were identified as DEmRNAs, DElncRNAs and DEmiRNAs, respectively (Supplementary Figure S2B).

In 2 m p.i. KC samples, total 102 upregulated and 1,291 downregulated mRNAs, 85 upregulated and 177 downregulated lncRNAs, and 5 upregulated and 38 downregulated miRNAs were found, while total 79 upregulated and 205 downregulated mRNAs, 69 upregulated and 32 downregulated lncRNAs, and 9 upregulated and 12 downregulated miRNAs were found in 3 m p.i. KC samples (Supplementary Figure S3A, Supplementary Table S2). The 111 (7.1%), 18 (5.2%) and 3 (4.9%) common DEGs were identified as DEmRNAs, DElncRNAs and DEmiRNAs, respectively (Supplementary Figure S3B).

### GO and KEGG Analyses of DElncRNAs and DEmiRNAs

**FIGURE 2:**
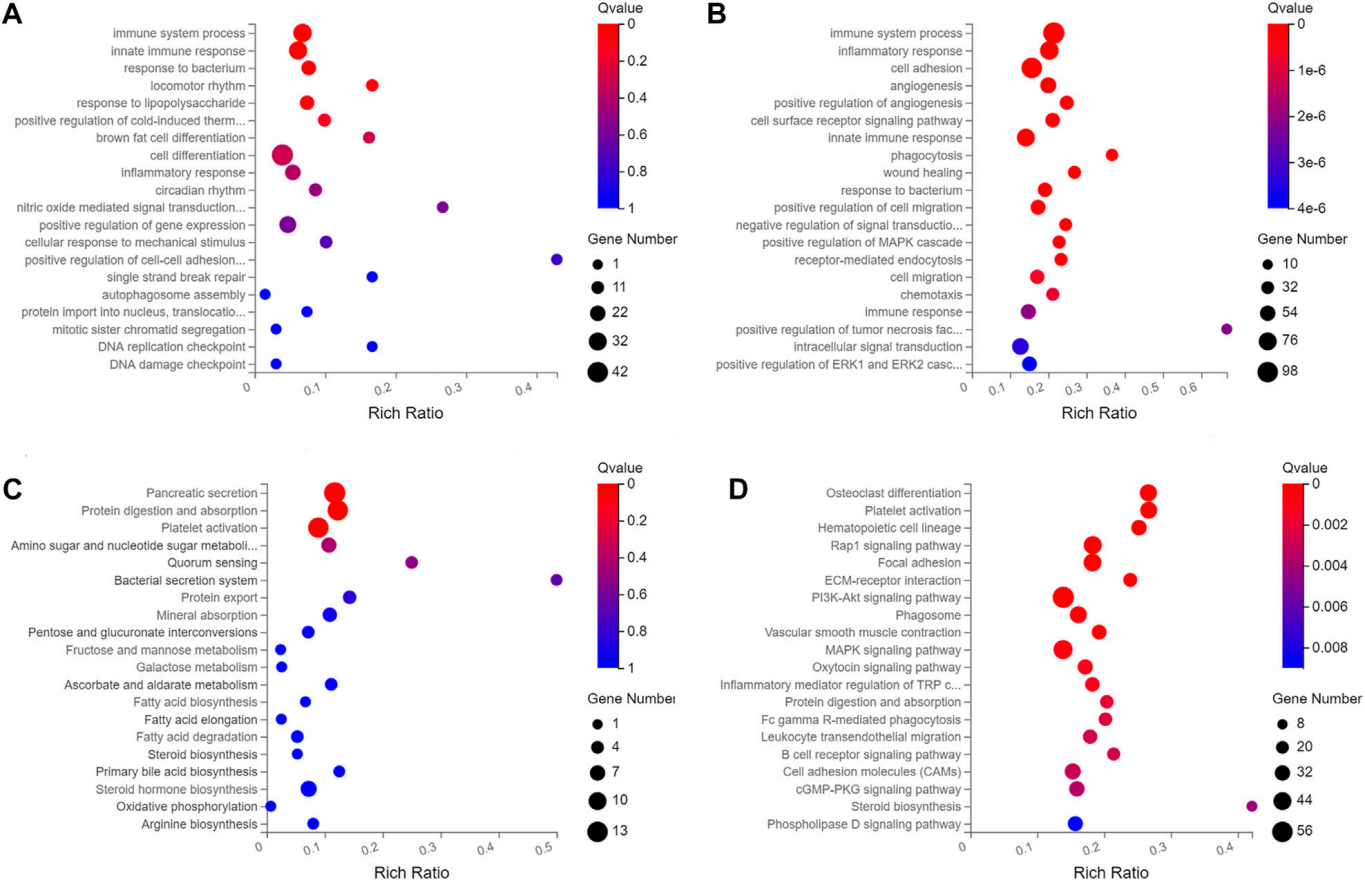
The top twenty enriched biological process and pathways of DElncRNAs and DEmRNAs in HCs at 2 m p.i. and 3 m p.i.. **(A)** Predicted biological process terms of DElncRNAs and DEmRNAs in HCs at 2 m p.i.. **(B)** Predicted biological process terms of DElncRNAs and DEmRNAs in HCs at 3 m p.i.. **(C)** Predicted pathways of DElncRNAs and DEmRNAs in HCs at 2 m p.i.. **(D)** Predicted pathways of DElncRNAs and DEmRNAs in HCs at 3 m p.i..

Of the 195 DEmiRNAs identified, the most was functionally annotated, such as miR-7a-5p, miR-223-3p, miR-22-3p, miR-146a-5p, miR-378a-3p, miR-467a-5p, miR-532-5p, miR-652-3p, miR-871-3p and miR-96-5p (Coffey et al., 2019; Ota et al., 2019; Chu et al., 2020; Du et al., 2020; Liang et al., 2020; Yan et al., 2020; Wang X. et al., 2021; Gajeton et al., 2021; Han et al., 2021; Kinoshita et al., 2021). Unlike the DEmiRNAs, most of the DElncRNAs were not annotated with unknown functions. Based on this, this study focused on the biological function of DElncRNAs, with the most enriched biological processes of DEmRNAs and DElncRNAs in HCs were immune system process, innate immune response, inflammatory response and cell adhesion (Figures 2A,B, Supplementary Table S4), and the enriched pathways included pancreatic secretion, protein digestion and absorption, fatty acid biosynthesis, ECM-receptor interaction and PI3K-Akt signaling pathway (Figures 2C, D, Supplementary Table S3).

In HSCs, the enriched biological processes included immune system process, adaptive immune response, negative regulation of lipid biosynthetic and receptor-mediated endocytosis (Supplementary Figures S4A, B, Supplementary Table S5), and the enriched pathways included T cell receptor signaling pathway, Th1 and Th2 cell differentiation, IL-17 signaling pathway, Toll-like receptor signaling pathway and NF-κB signaling pathway (Supplementary Figures S4C, D, Supplementary Table S4), indicating that HSCs may play an immune regulatory role after *E. multilocularis* infection.

While in KCs, the enriched biological processes included immune system process, acute-phase response, cellular response to IFN-β and exogenous drug catabolic process (Supplementary Figures S5 A, B, Supplementary Table S6), and the enriched pathways included complement and coagulation cascades, TNF signaling pathway, cytokine-cytokine receptor interaction, IL-17 signaling pathway and PPAR signaling pathway (Supplementary Figures S5C,D, Supplementary Table S5).

### LncRNA-mRNA-miRNA Networks

**FIGURE 3:**
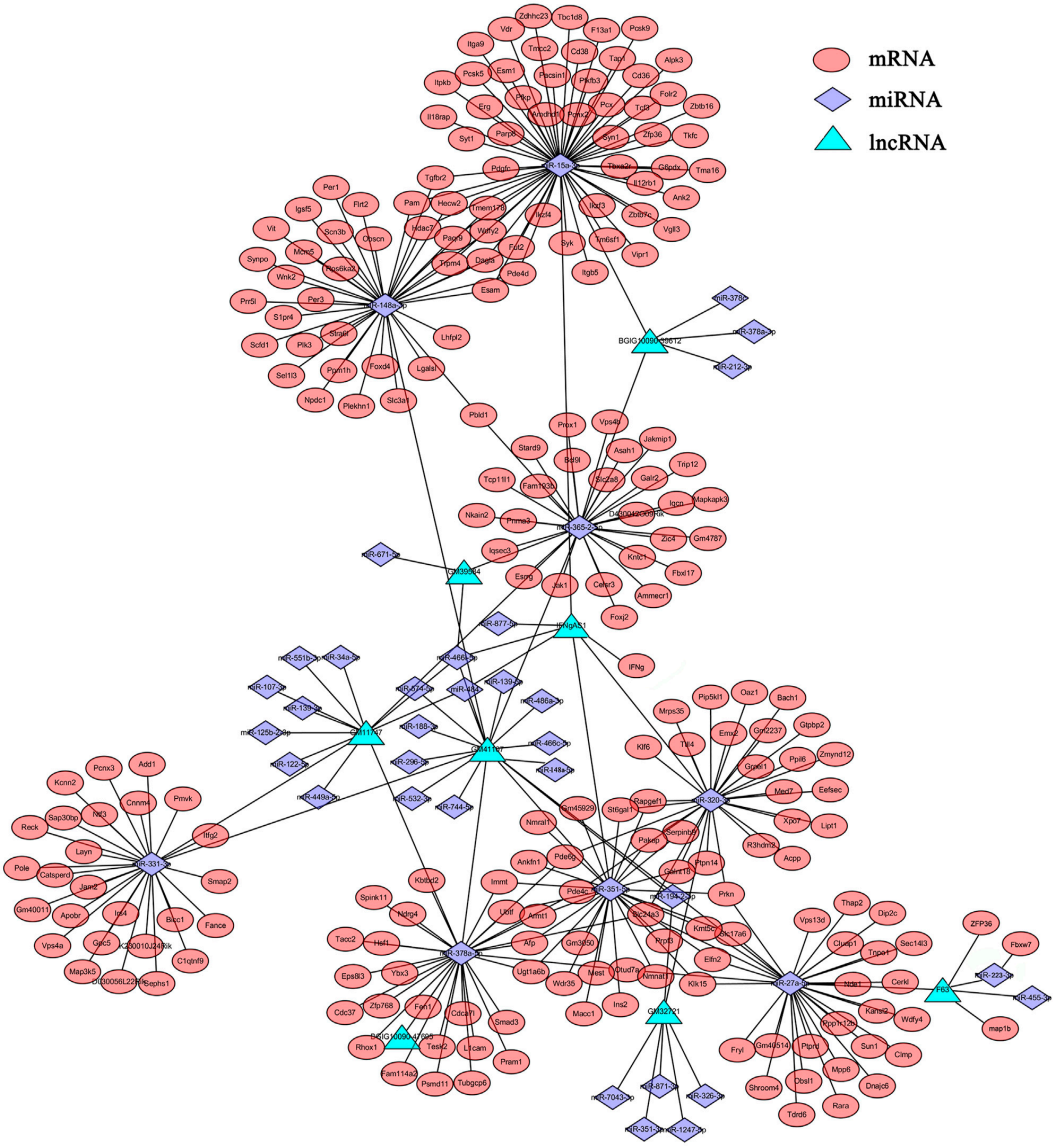
The lncRNA-mRNA-miRNA network. The relationships of lncRNAs-miRNAs-mRNAs were constructed based the results of predicted targets using Cytoscape software (v3.6.1). Different colors were used to show different genes, with green for DElncRNAs, purple for DEmiRNAs and red for DEmRNAs.

It has been demonstrated that lncRNAs function as miRNA “sponges”, which competitively suppress the activity of miRNAs (Alkan and Akgül, 2022). Since lncRNAs interact with miRNAs through miRNA Response Elements (MREs), combined using the free energy and score of RNAhybrid, miRanda and TargetScan databases, the potential MREs were predicted and 38 DEmiRNAs that putatively targeted 8 DElncRNAs were then identified (Supplementary Table S6). Based on these, partial lncRNA-mRNA-miRNA networks were obtained (Figure 3). In this network, some DElncRNAs were predicted to bind multiple DEmiRNAs, and DEmiRNAs were predicted to bind multiple DEmRNAs, such as F630028O10Rik (abbreviated as F63)-miR-223-3p-Fbxw7/ZFP36/map1b and F63-miR-27-5p-Tdrd6/Dip2c/Wdfy4. Considering these RNAs are differentially expressed in various cells, some important roles may play by these lncRNAs and miRNAs through these regulatory pathways, which provide some clues for further studies of DElncRNAs and DEmiRNAs.

### qRT-PCR Validation

**FIGURE 4:**
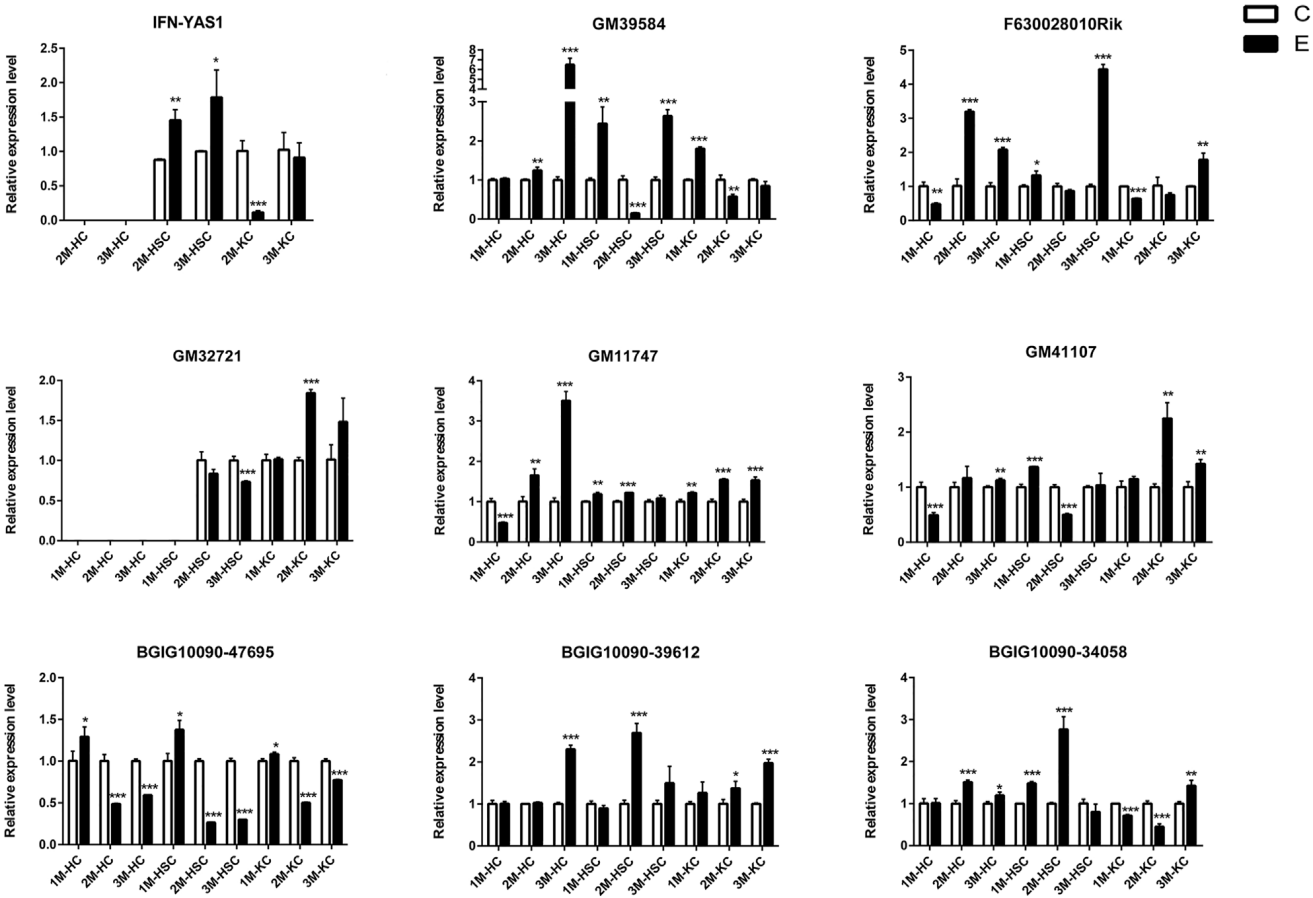
The relative expression levels of 9 lncRNAs in 2M-HC, 3M-HC, 2M-HSC, 3M-HSC, 2M-KC and 3M-KC were determined by qRT-PCR. The number of biological replicates for each experiment was 3 and the relative expression levels were normalized to the expression levels of GAPDH. Data are presented as means with SD. *p*-values were analyzed by Student’s *t*-test. ***p < 0.001, **p < 0.01 and *p < 0.05.

**FIGURE 5:**
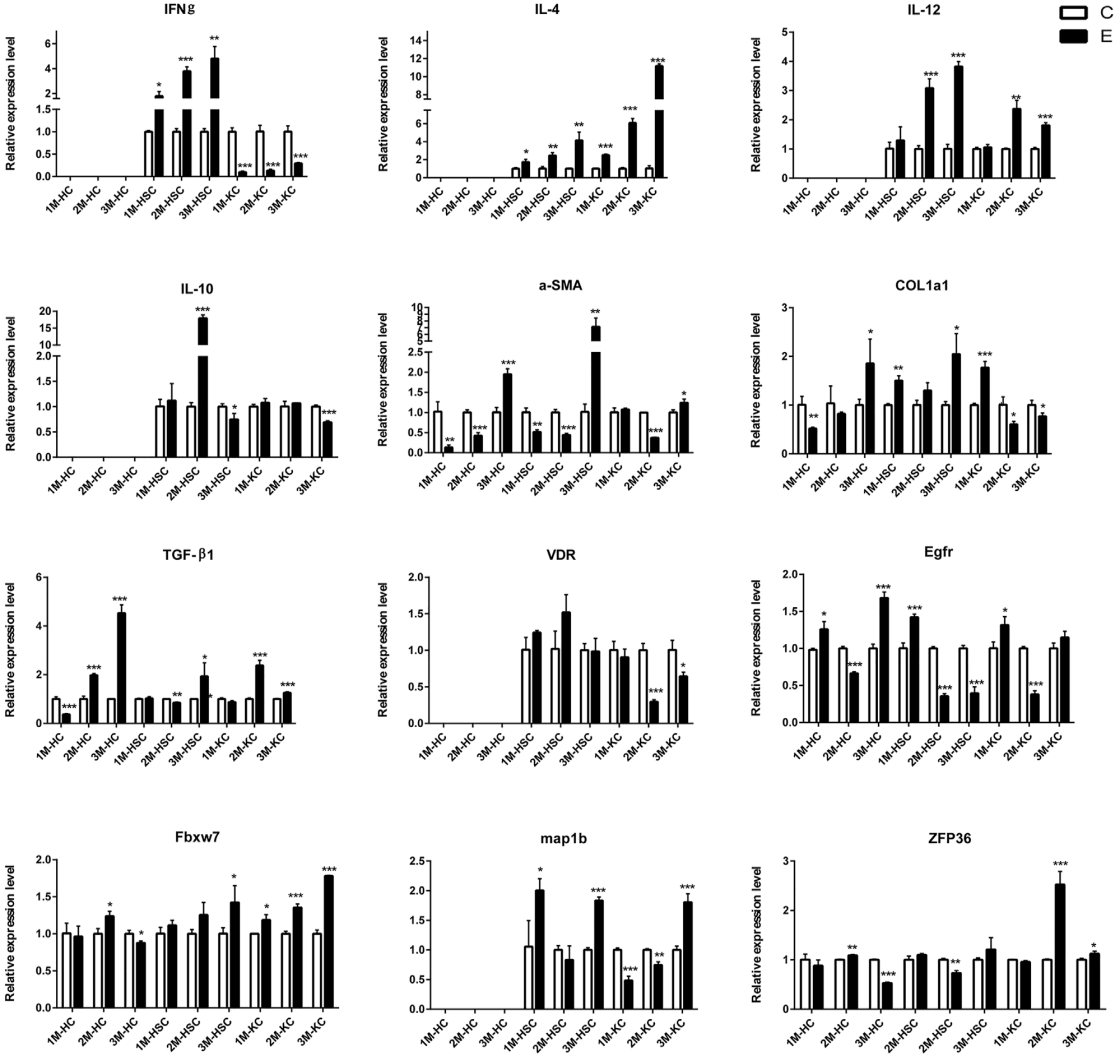
The relative expression levels of 12 mRNAs in 2M-HC, 3M-HC, 2M-HSC, 3M-HSC, 2M-KC and 3M-KC were determined by qRT-PCR. The number of biological replicates for each experiment was 3 and the relative expression levels were normalized to the expression levels of GAPDH. Data are presented as means with SD. *p*-values were analyzed by Student’s *t*-test. ***p < 0.001, **p < 0.01 and *p < 0.05.

**FIGURE 6:**
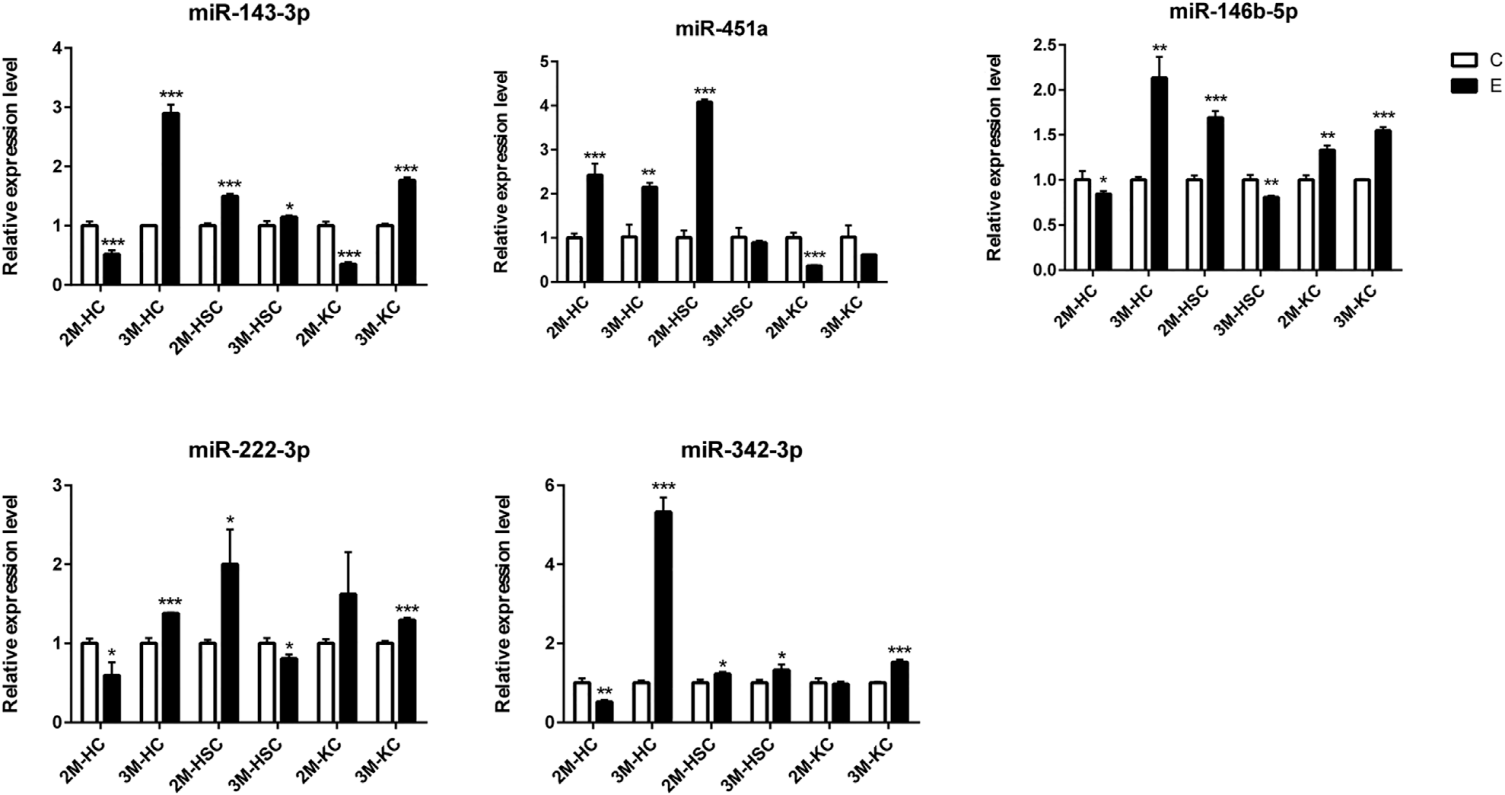
The relative expression levels of 5 miRNAs in 2M-HC, 3M-HC, 2M-HSC, 3M-HSC, 2M-KC and 3M-KC were determined by qRT-PCR. The number of biological replicates for each experiment was 3 and the relative expression levels were normalized to the expression levels of U6. Data are presented as means with SD. *p*-values were analyzed by Student’s *t*-test. ***p < 0.001, **p < 0.01 and *p < 0.05.

To verify the lncRNA-mRNA-miRNA networks and the data of RNA-seq, qRT-PCR was used to examine the expression of 9 DElncRNAs (IFNgAS1, GM39584, F63, GM32721, GM11747, GM41107, BGIG10090-47695, BGIG10090-39612 and BGIG10090-34058) (Figure 4), 12 DEmRNAs (IFN-γ, IL-4, IL-12, IL-10, a-SMA, COL1a1, TGF-β1, ZFP36, VDR, EGFR, Fbxw7 and map1b) (Figure 5) and 5 DEmiRNAs (miR-143-3p, miR-451a, miR-146b-5p, miR-222-3p and miR-342-3p) (Figure 6). The overall expression patterns of these DElncRNAs, DEmRNAs and DEmiRNAs between qRT-PCR and RNA-seq was consistent, suggesting the reliability of RNA-seq. It was worth mentioning that the expression of inflammation related genes (IFN-γ and IL-4) were remarkably upregulated in HSCs at 2 m p.i. and 3 m p.i. However, in KCs, the IFN-γ was remarkably downregulated and IL-4 still upregulated at 2 m p.i. and 3 m p.i. Additionally, the fibrosis related factors including α-SMA and Col1α1 showed a sharp increase in 3 m p.i., suggesting the persistence of inflammatory responses in the infection and the occurrence of fibrosis. Additionally, some lncRNA-mRNA pairs in the network were identified with a similar expression trend, such as GM39584-Egfr, F63-Fbxw7/map1b and IFNgAS1-IFN-γ, suggesting the potential regulatory role of these DElncRNAs.

**FIGURE 7:**
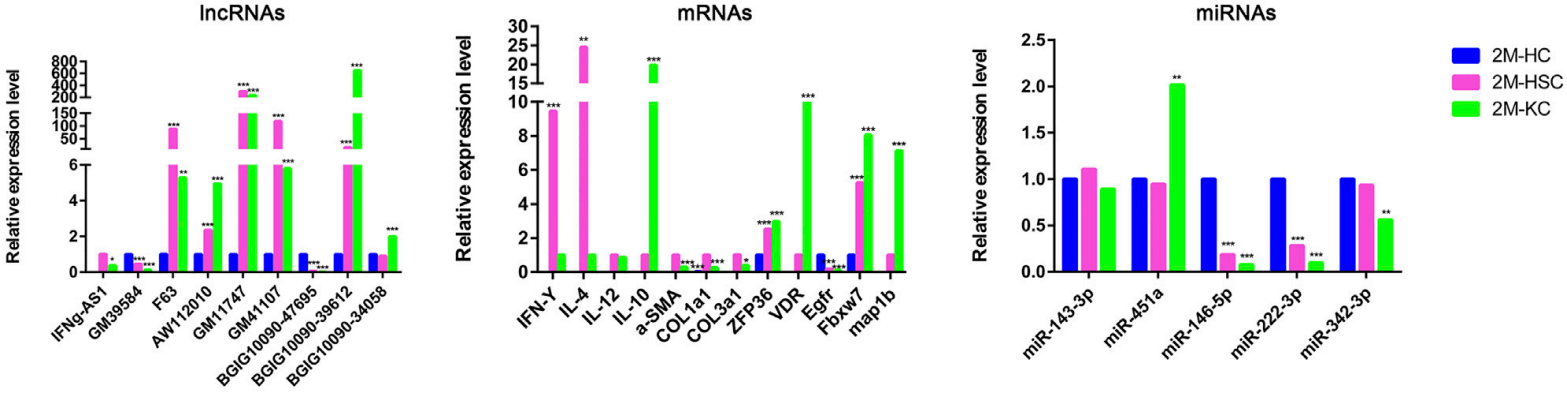
The relative expression level of DElncRNAs, DEmRNAs, and DEmiRNAs in different cells at 2 m p.i.. Sky blue represents the expression of genes in HCs, red and purple represents HSCs and KCs, respectively.

In addition, we identified some potential markers for HCs, HSCs and KCs (Figure 7). For instance, the expression of BGIG10090-47695, miR-146b-5p, miR-222-3p and EGFR was remarkably abundant in HCs comparing HSCs and KCs, while F63, GM41107 and GM32721 were remarkably abundant in HSCs, and BGIG10090-39612, map1b and VDR in KCs.

## Discussion

*E. multilocularis* is one of zoonotic tapeworms with public health concern. Elucidating the pathogenic mechanisms of the parasite in the liver will help us to take better prevention and treatment measures. It is known that HCs, HSCs, KCs together with others constitute the liver and play divergent roles. Exploring the specific biological processes of these cells after *E. multilocularis* infection will systematically clarify the pathogenic mechanisms. Therefore, the current study focuses on the transcriptomic maps of mRNA, miRNAs and lncRNAs expressed in HCs, HSCs and KCs in the liver after infection, which aims to provide clues for further investigation of the pathogenic mechanisms to better control AE.

The current results showed that, after the infection, the expression of a number of mRNAs, lncRNAs and miRNAs in HCs, HSCs and KCs was changed. One of our interests is the genes related to metabolism in HCs. For example, the F-box family member F-box- and WD repeat domain-containing 7 (Fbxw7) was up- and downregulated in 2 m p.i. and 3 m p.i., respectively. A previous study showed massive lipid deposition and increasing proliferation of hepatocytes in Fbxw7-deficient mice (Onoyama et al., 2011). Therefore, the changes in the expression of Fbxw7 may reflect the host’s response to eliminate the parasite in 2 m p.i. and the parasite’s strategy for persistent infection in 3 m p.i. Consistent with the lncRNA-mRNA-miRNA networks constructed, the qRT-PCR results suggested that Fbxw7 might be the potential target gene of miR-223-3p, which was regulated by F63 (lncRNA). It was reported that in *Cryptosporidium parvum* infection, cirs-7 was upregulated and promoted *C. parvum* propagation by regulating the miR-1270-RelA axis, which provided a control strategy against *C. parvum* infection (Yin et al., 2021). Therefore, elucidating the regulatory mechanism of F63-miR-223-3p-Fbxw7 may provide a potential target for the development of anti-*E. multilocularis* drugs. Another gene enriched in HCs, epidermal growth factor receptor (EGFR), was found to be highly expressed and was predicted to participate in the PI3K/AKT signaling pathway in 3 m p.i., suggesting an essential role in host defense against the infection. It is worth mentioning that EGFR was located upstream within 10 kb of GM39584 (lncRNA). Interestingly, their expression trend was consistent, suggesting that GM39584 play a *cis*-regulation role. It is worth exploring whether and how the EGFR-GM39584 axis plays a role during *E. multilocularis* infection.

For HSCs, we focused on the expression patterns of fibrosis-related genes, such as α-SMA, Col1α1 and Col1α3. As expected, *E. multilocularis* infection induced these genes to be upregulated, suggesting the tendency of liver fibrosis. Besides, Col1α1, one of the target genes of F63, was most enriched in HSCs. F63 acts as a competing endogenous RNA (ceRNA) for the miR-1231-5p/Col1α1 axis, involved in regulating post-spinal cord injury pyroptosis by activating the PI3K/AKT pathway (Xu et al., 2020). It is therefore hypothesized that F63 regulate the expression of Col1α1 indirectly and be involved in the apoptosis of hepatocytes. By analyzing the DEgenes of HSCs at 2 m p.i. and 3 m p.i., we found the inflammation-related pathways were enriched significantly, including cytokine-cytokine receptor interaction, chemokine signaling pathway, IL-17 signaling pathway, NF-kappa B signaling pathway, T cell receptor signaling pathway and Th1, Th2 and Th17 cell differentiation. Consistently, IFN-γ, IL-4 and IL-12 were remarkable upregulated in HSCs at 1 m p.i., 2 m p.i. and 3m p.i, suggesting that HSCs may be involved in immune regulation together with KCs. However, the level of IFN-γ in HSCs was higher than that of KCs and its expression trend in both cells was just opposite during the course of infection. Previous studies identified IFNgAS1 involved in IFN-γ-mediated host defense as an important regulator of IFN-γ expression and AW112010 promoted pro-inflammatory pathways by suppressing IL10 expression, which reduced the number of IFN-γ expression after knocked the expression of AW112010 (Petermann et al., 2019; Peng et al., 2020; Yang et al., 2020). In this work, the expression of IFN-γ in HSCs and KCs was positively correlated with AW112010 and IFNgAS1. Considering the similar expression pattern between IFNgAS1, AW112010 and IFN-γ, we guess that the similar role be played in HSCs after *E. multilocularis* infection. Therefore, whether the expression of IFN-γ is regulated by both AW112010 and IFNgAS1 is worthy of further exploration. Besides, it was reported that miR-155 positively regulates IFN-γ expression via the Tim-3 pathway in NK cells, and miR-29b/142-5p also induces IFN-γ upregulation by targeting DNMTs (Cheng et al., 2015; Yang Y. et al., 2018). In our work, we found that both miR-155 and miR-29b were downregulated expressed in HSCs at 3 m p.i., which provides a clue for investigation of the mechanism of IFN-γ regulation.

It is known that the liver resident macrophages, KCs, are the first line of defense against inflammation/infections in the liver. It has been shown that the activation of vitamin D receptor (VDR), expressed on KCs, decreases hepatic inflammation in diet-induced model of NASH (Dong et al., 2020). In this study, the expression of VDR was downregulated continuously, suggesting that KCs may be active against *E. multilocularis* infection. Previous studies identified that miR-125b and miR-351-5p involved in the pathological process by targeting VDR (Mohri et al., 2009; He et al., 2018). It is possible that miR-125b and miR-351-5p may play a similar regulatory role in KCs. Moreover, the expression of IFN-γ was decreased, while IL-4 and IL-10 were increased in KCs, implying that *E. multilocularis* infection cause a Th2 immune response rather than Th1 immune response. Additionally, the KEGG pathway analysis revealed that the DEgenes were significantly enriched in the IL-17 signaling pathway, TNF signaling pathway and cytokine-cytokine receptor interaction. We found some metabolism-related pathways were also enriched, such as ascorbate and aldarate metabolism, steroid hormone biosynthesis, glycine, serine and threonine metabolism, tryptophan metabolism, biosynthesis of secondary metabolites, arginine biosynthesis and fatty acid biosynthesis, indicating that KCs also play a role in metabolism regulation during *E. multilocularis* infection.

In summary, the present study revealed the transcriptomic maps of mRNA, miRNAs and lncRNAs expressed in HCs, HSCs and KCs during *E. multilocularis* infection. Additionally, by integrate analyzing the RNA-seq data, we found some potential regulatory axis, such as F63-miR-223-3p-Fbxw7, GM39584-EGFR, F63-miR-1231-5p-Col1α1, IFNgAS1/AW112010-IFN-γ, and miR-125b/miR-351-5p-VDR. The future studies need to insight into clarify these regulatory axis and provide potential treatment targets for AEs.

## Data Availability

The original contributions presented in the study are publicly available. This data can be found here: PRJNA732233 and PRJNA770143.

## Supplementary Material

Supplementary Figure 1 The expression of cell markers in HCs, HSCs and KCs.

Supplementary Figure 2 Comparisons of DElncRNAs, DEmRNAs and DEmiRNAs differentially expressed in HSCs at 2 m p.i. and 3 m p.i.. **(A)** The number of DElncRNAs, DEmRNAs and DEmiRNAs at two infection stages. Sky blue represents the total number of DEgemes, red and violet represents upregulated and downregulated genes, respectively. **(B)** Veen diagrams showing the common and unique DElncRNAs, DEmRNAs and DEmiRNAs at two infection stages.

Supplementary Figure 3 Comparisons of DElncRNAs, DEmRNAs and DEmiRNAs differentially expressed in KCs at 2 m p.i. and 3 m p.i.. **(A)** The number of DElncRNAs, DEmRNAs and DEmiRNAs at two infection stages. Sky blue represents the total number of DEgemes, red and purple represents upregulated and downregulated genes, respectively. **(B)** Veen diagrams showing the common and unique DElncRNAs, DEmRNAs and DEmiRNAs at two infection stages.

Supplementary Figure 4 The top twenty enriched biological process and pathways of DElncRNAs and DEmRNAs in HSCs at 2 m p.i. and 3 m p.i.. **(A)** Predicted biological process terms of DElncRNAs and DEmRNAs in HSCs at 2 m p.i.. **(B)** Predicted biological process terms of DElncRNAs and DEmRNAs in HSCs at 3 m p.i.. **(C)** Predicted pathways of DElncRNAs and DEmRNAs in HSCs at 2 m p.i.. **(D)** Predicted pathways of DElncRNAs and DEmRNAs in HSCs at 3 m p.i..

Supplementary Figure 5 The top twenty enriched biological process and pathways of DElncRNAs and DEmRNAs in KCs at 2 m p.i. and 3 m p.i.. **(A)** Predicted biological process terms of DElncRNAs and DEmRNAs in KCs at 2 m p.i.. **(B)** Predicted biological process terms of DElncRNAs and DEmRNAs in KCs at 3 m p.i.. **(C)** Predicted pathways of DElncRNAs and DEmRNAs in KCs at 2 m p.i.. **(D)** Predicted pathways of DElncRNAs and DEmRNAs in KCs at 3 m p.i..
