# Supplementary figures and images for: Integrative Analysis of RNA Expression and Regulatory Networks in Mice Liver Infected by Echinococcus multilocularis
Source: Front Cell Dev Biol. 2022 Mar 24;10:798551. doi: 10.3389/fcell.2022.798551 (PMC8989267; doi:10.3389/fcell.2022.798551)

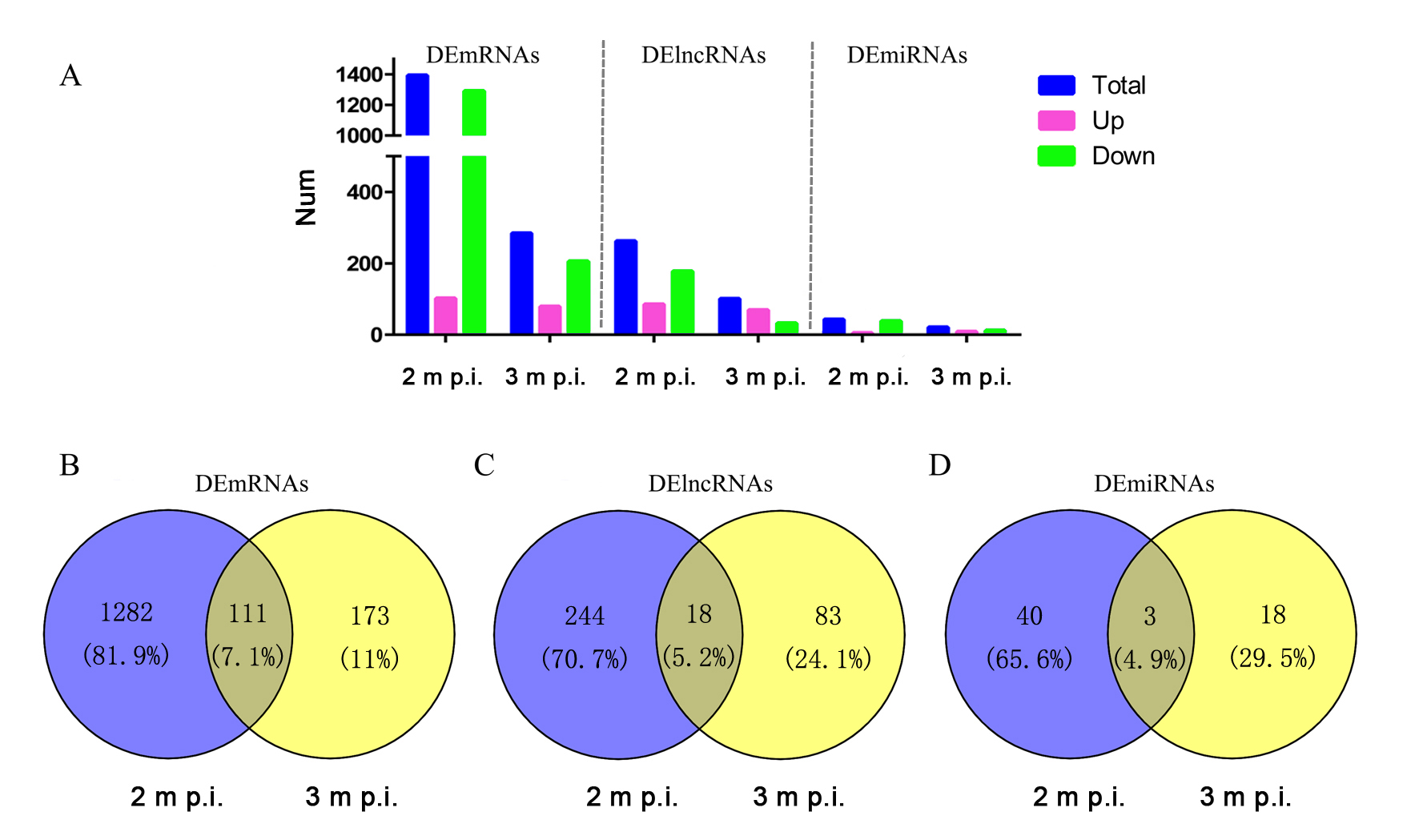

Supplement: Supplementary file 3 [file Image3.JPEG]

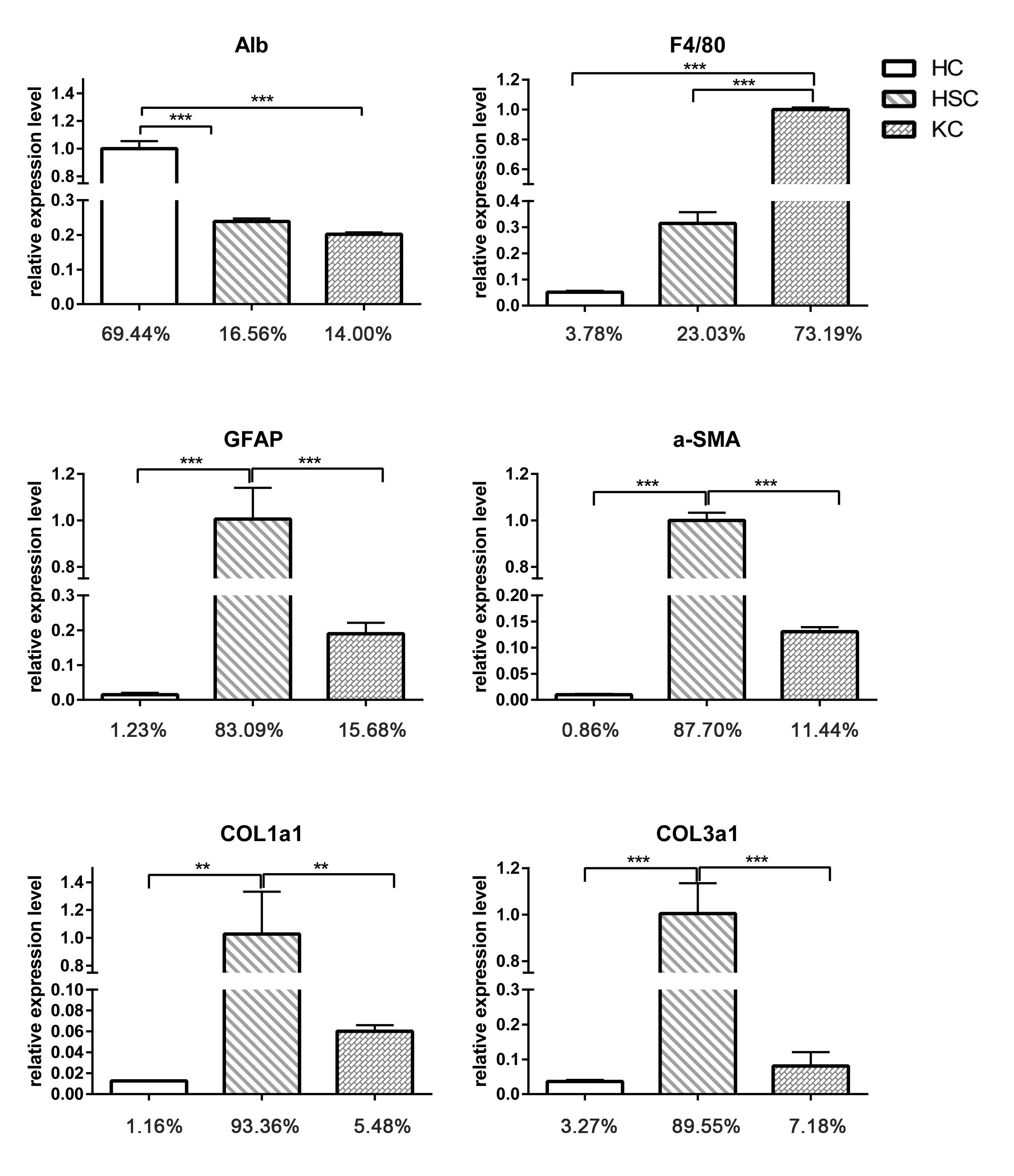

Supplement: Supplementary file 5 [file Image1.JPEG]

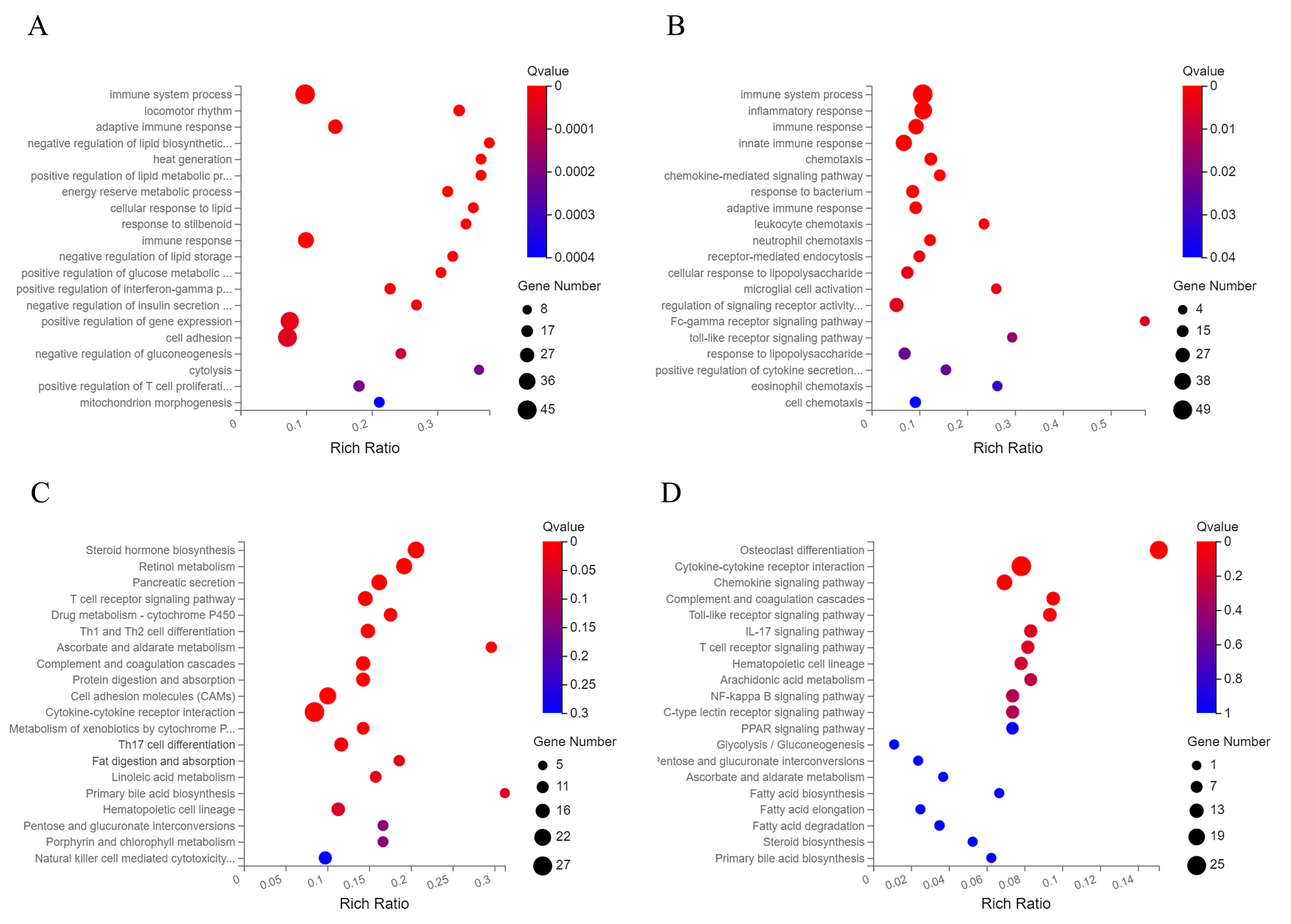

Supplement: Supplementary file 6 [file Image4.JPEG]

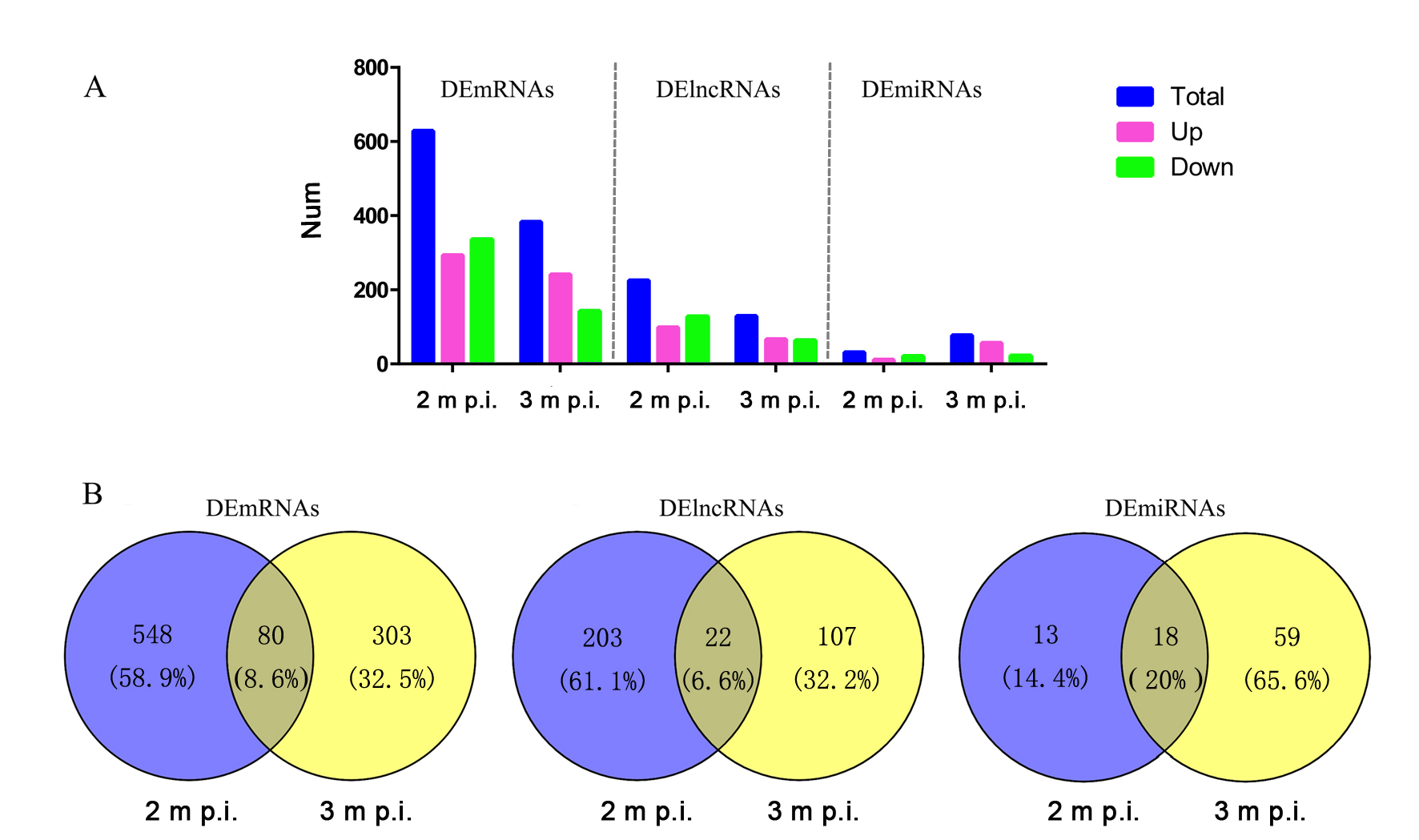

Supplement: Supplementary file 8 [file Image2.JPEG]

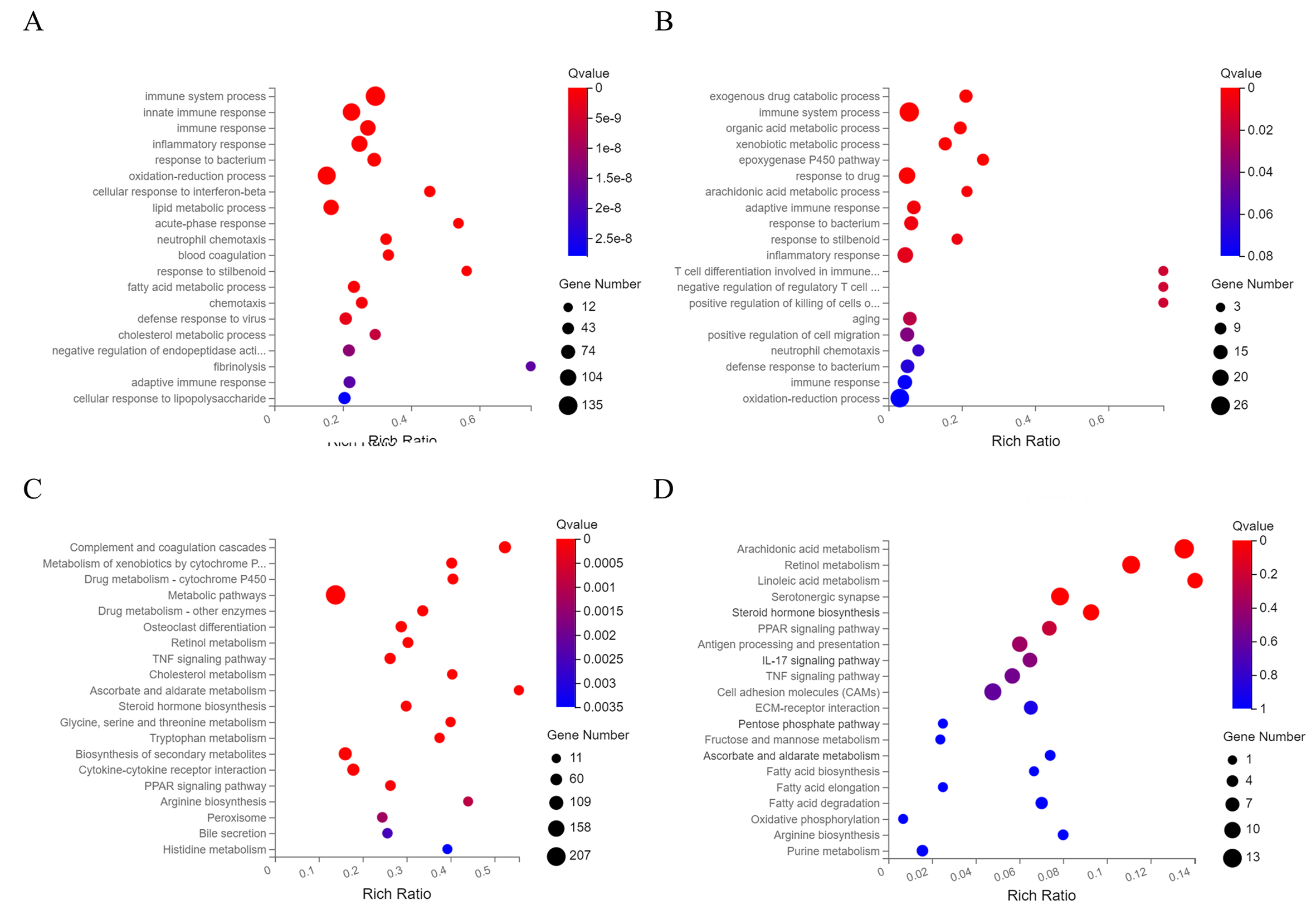

Supplement: Supplementary file 9 [file Image5.JPEG]
